# Multiple symmetric lipomatosis (Madelung disease) presenting as cervical lipomatous mass in a middle-aged male with alcohol use in Nepal: A Case Report

**DOI:** 10.1186/s12893-026-03789-0

**Published:** 2026-05-02

**Authors:** Suzita Hirachan, Adheesh Bhandari

**Affiliations:** 1 Department of General Surgery, Institute of Medicine, Tribhuvan University Teaching Hospital, Kathmandu, Nepal; 2 Department of General Surgery, Breast and Thyroid Unit, Primera Hospital, Kathmandu, Nepal

**Keywords:** Madelung disease, Treatment, Lipomatosis, Multiple Symmetrical

## Abstract

Multiple symmetric lipomatosis (MSL), or Madelung disease, is a rare disorder characterized by symmetrical, non-encapsulated adipose tissue deposition, predominantly involving the neck and upper trunk. It is strongly associated with chronic alcohol consumption.

We report a 33-year-old male with a history of regular alcohol intake presenting with a progressively enlarging cervical mass. Imaging revealed bilateral, symmetrical lipomatous deposits consistent with MSL. The patient underwent surgical excision under general anesthesia with satisfactory cosmetic and functional outcomes.

This report highlights the clinical presentation, diagnostic approach, and management of MSL, emphasizing the importance of early recognition. A short-term follow-up showed no recurrence; however, long-term surveillance and alcohol cessation remain essential to reduce recurrence risk.

## Introduction

Madelung disease (MSL) is a rare disorder characterized by the proliferation of unencapsulated adipose tissue in a symmetrical distribution, predominantly affecting the cervical, shoulder, and upper trunk regions [1, 2]. Madelung’s disease was first described in 1846 by Brodie, and in 1888 [3] it was also described by Madelung. This condition is more prevalent in middle-aged males with a history of alcohol consumption. Its pathogenesis is not fully understood, but it is believed to involve mitochondrial dysfunction and abnormal adipocyte proliferation [4].

The clinical presentation varies from cosmetic deformity to compressive symptoms, depending on the size and location of the lipomatous masses. The diagnosis is primarily radiological, with support from histopathological examinations. Treatment options include surgical excision and liposuction with management of associated metabolic derangements.

## Case presentation

**Fig. 1:**
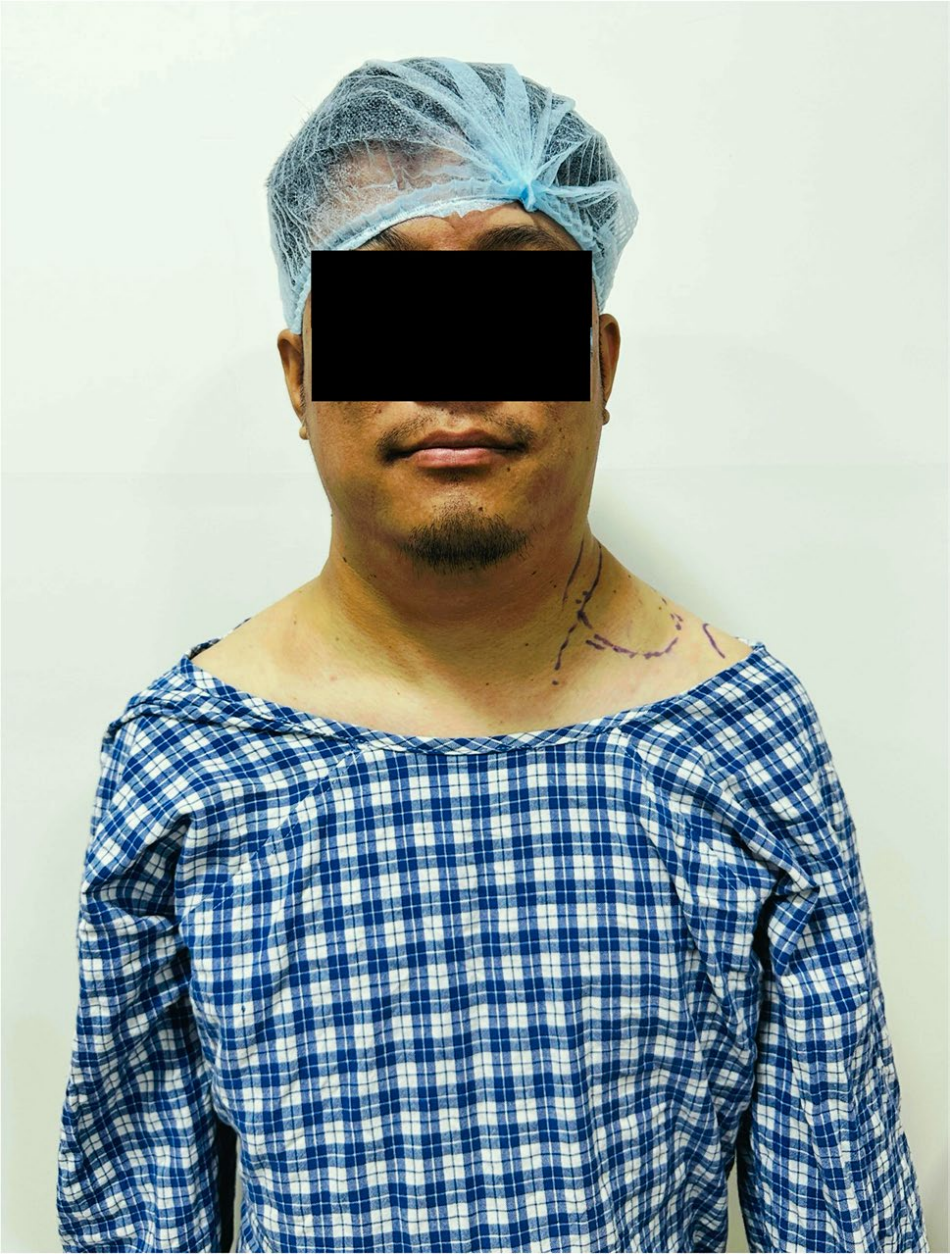
A photograph of a 33-year-old man, showing large, symmetrical, protruding mass lesions at the anterior aspects of the left supraclavicular area

A 33-year-old married male with a history of alcohol consumption approximately every three days presented with a gradually enlarging swelling over the left lateral neck that had persisted for 20 days (Fig. 1). The swelling was soft, diffuse, and non-tender, measuring approximately 8 × 5 cm over the posterior triangle, with no associated systemic symptoms, such as fever, pain, or shortness of breath. The patient denied any recent trauma or comorbidities. On general examination, he appeared well oriented with no signs of pallor, jaundice, cyanosis, clubbing, or edema. Systemic examination was unremarkable, with no palpable cervical lymph nodes; the overlying skin appeared normal.

### Additional patient characteristics

- Body mass index (BMI): 27.4 kg/m² (overweight).
- No clinical features of generalized obesity or Cushingoid appearance.

## Distribution of lesions

**Fig. 2:**
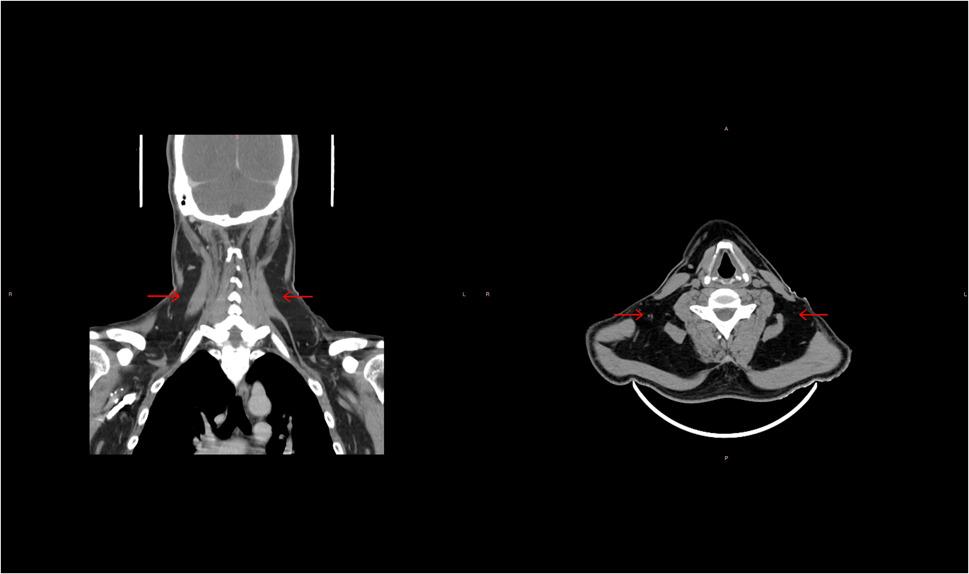
Arrows indicate symmetrical, non-encapsulated fat-attenuated deposits consistent with Madelung disease

Contrast-enhanced CT demonstrated bilateral, symmetrical, non-encapsulated fat deposition 99 × 66 × 202 mm in the posterior cervical region, extending to the supraclavicular areas, confirming the characteristic pattern of MSL. (Fig. 2, arrows added to highlight lesions). No significant deposits were observed in the abdomen or lower body.

Laboratory tests revealed elevated triglyceride levels (420 mg/dL) and mild increases in liver enzymes (SGPT, 65 U/L; SGOT, 47 U/L), while all other parameters remained within normal limits. Serological test results for HIV, HBsAg, and hepatitis C virus (HCV) were negative. Echocardiography and electrocardiography revealed normal cardiac function, and thyroid function tests were unremarkable, indicating no systemic metabolic or cardiac abnormalities.

**Fig. 3:**
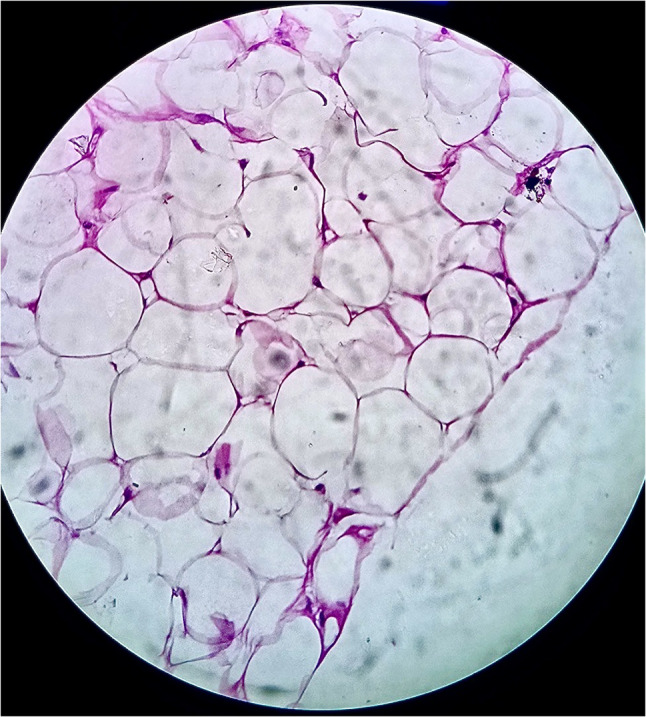
Photomicrograph of Fine needle aspiration cytology 40x shows lobules of mature adipose tissue encapsulated by a thin fibrous capsule, separated by thick fibrous bands with few intervening capillaries and no features of atypia

Further investigations included ultrasonography of the neck, which revealed a lipomatous lesion in the posterior triangle. On fine-needle aspiration cytology (FNAC), sections showed lobules of mature adipose tissue encapsulated by a thin layer of fibrous tissue and separated by thick fibrous bands. A few intervening capillaries are also observed. No atypical features were noted (Fig. 3).

Based on the clinical, radiological, and cytological findings, a diagnosis of Madelung disease was established.

## Surgical technique

The patient underwent surgical excision under general anesthesia.

**Fig. 4:**
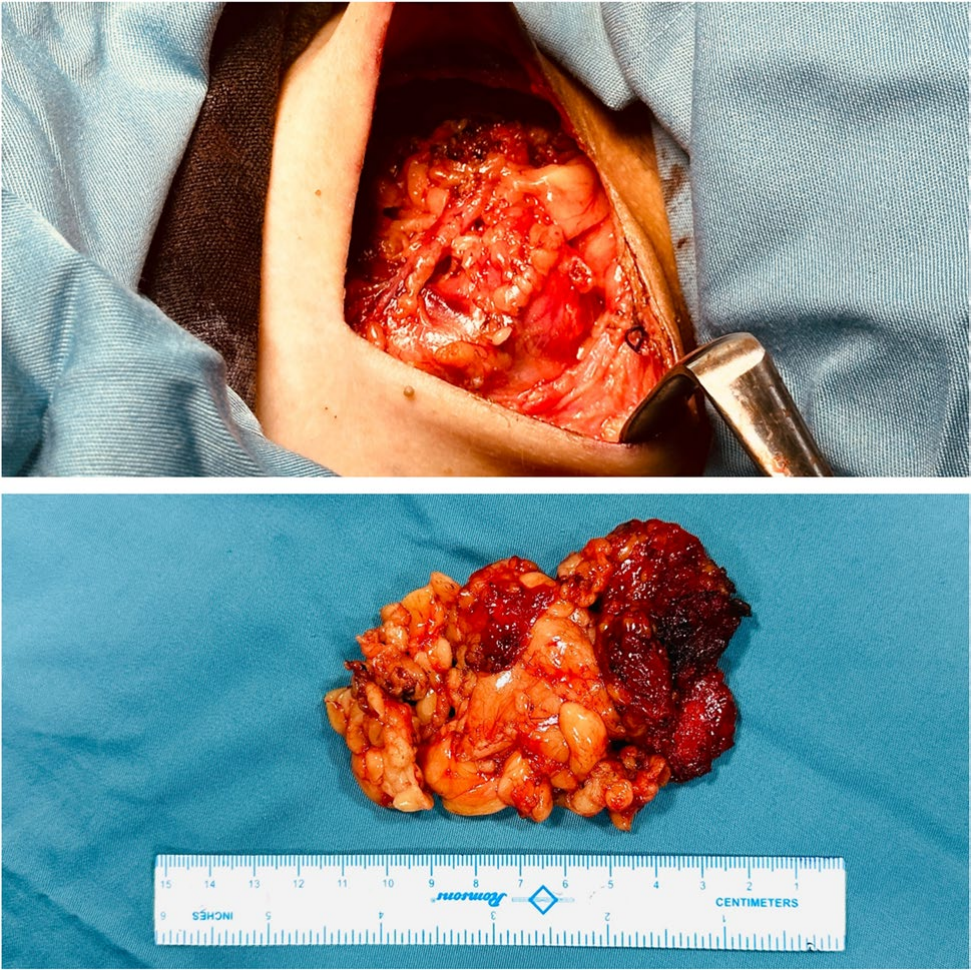
Showing the left supraclavicular lump

- Preoperative planning included marking of the lesion boundaries based on CT findings.
- Tumescent (Klein) solution was not used.
- A transverse cervical incision was made.
- Careful layer-by-layer dissection was performed.
- The adipose tissue was non-encapsulated and diffusely infiltrative, requiring meticulous separation.
- Critical neurovascular structures, including the spinal accessory nerve and major vessels, were preserved (Fig. 4).

- Hemostasis was secured, and a suction drain was placed.

Histopathology confirmed benign lipomatosis. At the 1-month follow-up, there was no recurrence, and the cosmetic appearance was satisfactory.

## Postoperative course and patient perspective

The postoperative period was uneventful. The patient reported significant improvement in neck contour and satisfaction with the cosmetic outcome.

He was counseled extensively regarding:

- Alcohol cessation.
- Lifestyle modification.
- Risk of recurrence.

At 1-month follow-up:

- No recurrence observed.
- Patient compliance with alcohol reduction was partial.

Long-term follow-up is planned, as recurrence is common in MSL, especially in patients who continue alcohol consumption.

## Discussion

Multiple symmetric lipomatosis (MSL), also known as Madelung disease, is a rare disorder characterized by symmetrical, non-encapsulated adipose tissue accumulation, predominantly affecting the neck, shoulders, and upper trunk. It most commonly occurs in middle-aged men with a strong association with chronic alcohol consumption [4]. 

Although initially described in Mediterranean populations, increasing evidence suggests that MSL is also present in Asian populations, where it may be underdiagnosed. Several cases have been reported from countries such as China and Korea, demonstrating similar clinical patterns, including symmetrical fat deposition and metabolic disturbances [2, 5]. These findings support the global relevance of this condition.

Painless subcutaneous unencapsulated fatty tissue overgrowth primarily affects the head, neck, and upper torso in a symmetrical pattern, with subsequent progression to the upper extremities [6]. The disorder occurs more frequently in Mediterranean populations and individuals of Mediterranean descent, with an estimated incidence of 1:25,000 in Italy [7]. Most affected individuals are men aged 40–59 years with a history of chronic excessive alcohol consumption, particularly red wine [7–9]. Approximately 90% of patients present with concurrent liver cirrhosis [1]. Type 1 phenotype predominates and is characterized primarily by cervical fat deposition [8, 10]. 

The pathogenesis remains elusive, but is closely linked to alcohol-induced mitochondrial dysfunction, leading to abnormal adipocyte proliferation. It is often associated with metabolic syndromes, including hyperlipidemia, as was observed in this patient. Adipogenesis reflects active adipose tissue proliferation, rather than merely resulting from an energy surplus. The proposed mechanisms include respiratory chain dysfunction along with mitochondrial DNA deletions and mutations [11]. Lipomatous fat deposits may arise from functionally impaired brown adipose tissue [7]. Diagnosing MSL can be challenging in certain cases and is frequently overlooked owing to the high prevalence of obesity. These patients often exhibit features of metabolic syndrome, including type 2 diabetes mellitus, glucose intolerance, hypertension, hyperlipidemia, and hyperuricemia. Alcohol-related complications such as hepatopathy, macrocytic anemia, and peripheral neuropathy also commonly occur in patients with MSL [12]. In 1984, Enzi categorized MSL into two types based on fat deposition patterns. Type I MSL presents as symmetric, well-circumscribed fat masses protruding from characteristic anatomical sites, including the parotid glands (hamster cheeks), cervical region (horse collar), posterior neck (buffalo hump), submental area (Madelung collar), shoulders, supraclavicular triangle, and the proximal upper limbs. Type II MSL features diffuse subcutaneous fat accumulation, primarily affecting the abdomen and thighs, resulting in a distribution pattern similar to that of generalized obesity [4]. In 1991, Donhauser et al. [13] expanded Enzi’s classification by introducing type III (gynecoid), characterized by predominant fat deposition in the pelvic region. The present case involved a middle-aged male with chronic alcohol abuse, exhibiting lipomatosis confined to the anterior and posterior neck, parotid region, and upper back, consistent with type I MSL.

Imaging modalities, such as ultrasound, CT, and MRI, are crucial for diagnosis, revealing characteristic non-encapsulated, fat-attenuated lesions. Histopathological examination confirmed proliferation of mature adipocytes without atypia. Differential diagnoses of MSL include obesity, Cushing syndrome, angiolipomatosis, encapsulated fibromas, neurofibromatosis, myxoid liposarcoma, lymphoma, salivary gland disease, Frölich syndrome, and HIV-associated lipomatosis [1, 14]. Recent molecular studies employing next-generation sequencing have revealed greater genetic diversity in patients with Madelung’s disease than in healthy controls [2]. 

Treatment primarily involves surgical removal or liposuction, particularly for large or symptomatic masses. The management of metabolic abnormalities and alcohol cessation can help prevent recurrence. The current management of Madelung’s disease remains limited to palliative measures, such as surgical debulking, liposuction, or injection lipolysis [2, 15]. Addressing metabolic dysfunction and excess weight may provide clinical benefits. Although alcohol cessation and weight reduction can help mitigate symptoms, these interventions often fail to halt disease progression [2, 8]. Nearly 63% of patients develop recurrence following adipose tissue resection, with liposuction cases showing recurrence rates as high as 95% [8, 16]. Post-injection lipolysis often leads to fibrosis and adhesions, complicating subsequent surgical interventions for recurrent lesions [11]. Although the relapse rates remain high, surgical approaches can achieve functional and cosmetically acceptable outcomes [17]. Current pharmacotherapeutic options for Madelung’s disease remain ineffective, as demonstrated by the lack of clinical efficacy of salbutamol-induced adrenergic lipolysis in large-scale trials [8, 18]. 

## Conclusion

This case highlights the importance of recognizing Madelung disease in patients presenting with symmetrical cervical masses, particularly in the context of alcohol use. Detailed imaging, careful surgical planning, and long-term follow-up are essential for optimal management. Addressing modifiable risk factors, especially alcohol consumption, plays a critical role in reducing recurrence.

## Consent

Written informed consent was obtained from the patient for publication and any accompanying images. A copy of the written consent is available for review by the Editor-in-Chief of this journal on request.

## Data Availability

All datasets generated and/or analyzed during the current study are publicly available upon reasonable request.
